# Anti-BRAF/anti-MEK targeted therapies for metastatic melanoma patients during the COVID-19 outbreak: experience from an Italian skin cancer unit

**DOI:** 10.2217/fon-2020-0997

**Published:** 2021-02-03

**Authors:** Pietro Quaglino, Paolo Fava, Matteo Brizio, Elena Marra, Marco Rubatto, Martina Merli, Luca Tonella, Simone Ribero, Maria Teresa Fierro

**Affiliations:** ^1^Dermatology Clinic, University of Turin, Turin, 10124, Italy

**Keywords:** advanced metastatic melanoma, COVID-19, targeted therapies

Although melanoma has been increasing at a constant rate in the past few decades, mortality has been low [1], and only a small percentage of patients have developed metastatic disease [2]. The presence of *BRAF* somatic mutations in >50% of cases with the constitutive activation of the MAPK pathway has led to the development of a targeted therapy (TT) with anti-BRAF/anti-MEK inhibitors. TT includes three drug combinations: dabrafenib/trametinib, vemurafenib/cobimetinib and encorafenib/binimetinib. In randomized clinical trials, these combinations have proven to significantly improve disease outcome, with response rates of up to 70% and a median survival rate of >2 years [3]. Recently published data from the 5-year pooled analysis of COMBI-d and COMBI-v trials of dabrafenib and trametinib, report an overall survival rate of 34% [4]. The same drugs have also been used in an adjuvant setting in stage III disease-free patients [5].

COVID-19 is a pandemic disease caused by a newly identified β-coronavirus (SARS-CoV-2) and presents predominantly as a respiratory infection, with the most common symptoms being fever, fatigue and a dry cough [6]. The clinical course of the infection can be complicated further by the development of pneumonia, which can lead to acute hypoxic respiratory failure and/or death [7]. Higher mortality rates are related to COVID-19 patients who are elderly, have previously had cancer and those with comorbidities such as hypertension and diabetes [8]. A report has shown that active cancer treatments do not represent a risk factor for COVID-19 infection. However, compared with the nononcologic population, the prognosis of infected cancer patients appears to be worse [9]. As of September 2020, the total number positive cases in Italy was 293,025 with 35,658 deaths [10]. Our university hospital, located in Piedmont (northwestern Italy) observed more than 15,000 cases, with a daily increment peak up to 4.2%.

Recommendations from the European Society for Medical Oncology stress the importance of testing before treatment in COVID-19 patients. They state that decisions for treatment initiation or continuation must be discussed with both COVID-19-positive and -negative patients, depending on their symptoms [11]. The Italian Association of Medical Oncology has also suggested evaluating each patient on a case-by-case basis and consider whether to continue treatment, depending on the biological features of the tumor, the patient's clinical status and the potential risks of COVID-19 infection [12]. The importance of the doctor–patient relationship to prevent independent decision-making of treatment discontinuation and to properly manage side effects was underlined in a recent commentary by Conforti *et al*. [13].

We report our experience in the management of melanoma patients undergoing TT treatment and discuss whether such treatments should be delayed, withdrawn or started.

Preclinical results on animals infected with coronavirus suggest that viral infection replication upregulates the MAPK pathway (BRAF/MEK/ERK), which in turn favors viral replication and host cell apoptosis. Murine coronavirus mouse hepatitis virus induces the activation of the MAPK pathway, whereas the inhibition of the same pathway significantly impairs viral replication [14]. Data on porcine delta-coronavirus [15] and avian coronavirus infectious bronchitis virus [16] suggest that MEK/ERK signaling plays a major role in viral replication. Although these results were obtained with animal coronavirus and not with the new COVID-19 virus, hypotheses can be made of the potential important implications of the data in the frame of the ongoing COVID-19 pandemic. TT induces an inhibition of the MAPK pathway in metastatic melanoma cells that harbor the *BRAF* mutation, while also promoting a paradoxical activation of the same pathway in *BRAF* wild-type cells [17]. This phenomenon, which represents the molecular basis for the development of TT-related adverse events, could potentially favor viral replication in infected, metastatic melanoma patients who have been treated with an anti-BRAF inhibitor. On the other hand, data from clinical trials support the fact that treatment discontinuation leads to the development of disease progression, even in patients with sustained responses [18]. This is different from patients treated with checkpoint inhibitors; the KeyNote-006 study showed that a complete/partial response or stable disease maintaining the response was clearly achieved in 78% of patients who completed 2 years of pembrolizumab [19] and implies a different patient management strategy.

Although there have been some reports on the management strategy during COVD-19 outbreak in melanoma patients treated by immune checkpoint inhibitors [20], there are no data available in patients treated with targeted therapies in a COVID-19 real-life setting.

At the beginning of March 2020, there were 67 advanced metastatic melanoma patients on TT at our center: 58 with dabrafenib/trametinib, six with vemurafenib/cobimetinib and three with encorafenib/binimetinib. There were also 23 patients who were in adjuvant treatment for disease-free stage III exclusively with dabrafenib/trametinib. At the beginning of the COVID-19 outbreak, we decided to maintain treatment in all patients due to the available clinical data on the increased relapse risk in patients discontinuing TT. However, because preclinical data suggested a relationship between viral infection and upregulation of the MAPK pathway, the monitoring of the signs and symptoms in both patients and their family members was carefully undertaken to detect early potential COVID-19 infection. High body temperature as a symptom was challenging because of the difficulties in discriminating between viral- and treatment-related illness (i.e., fever represents the most frequent adverse events in patients treated with the dabrafenib/trametinib combination) [3,4,17]. In addition, during the observed period, seven patients started a new TT treatment: five dabrafenib/trametinib, one vemurafenib/cobimetinib and one encorafenib/binimetinib).

Before COVID-19, patients were examined monthly at our clinic, and expected drug doses for the next treatment interval were supplied. Because patients sometimes discontinued their treatment due to side effects, it could be possible that they saved some extra doses at home.

When the COVID-19 outbreak started, all patients were informed of the contagion risk and the possibility of continuing treatment; they were also asked how many doses they had available at home. In case of no drug-related or melanoma-related symptoms, scheduled hospital visits were postponed by 1–3 weeks, and saved drug doses were used instead of new ones.

A strict triage procedure was put in place at the hospital entrance. All patients were screened by a nursing team for symptoms. Their temperature was checked before admittance, and they were asked whether they had been in contact with symptomatic or COVID-19-positive patients and/or family members. Patients who could enter were invited to carefully disinfect their hands and provided with rubber gloves and surgical masks. In the onco-dermatological service, each patient was assigned to a separate area, with adequate distance and with no accompanying people.

Due to the pandemic, older adults, patients with comorbidities and those who could not get to the hospital struggled to access their medication. Instead, their medications were sent directly to their home through a hospital service; alternatively, the patients' family members could pick them up from the hospital. For patients who were unable to reach the hospital, blood tests including blood cell count, liver and kidney function tests, glucose, lactate dehydrogenase and electrolytes, including magnesium, were prescribed, and the patients were asked to send the results to our service by mail.

At the time of writing, no patients under TT developed COVID-19 infection. Patients whose referred body temperature was >38.5° were asked about other symptoms (cough, dyspnea, dysgeusia) and whether they had potentially been in contact with any known COVID-19-positive people or family members with COVID-19-related symptoms. They were suggested to interrupt anti-BRAF/anti-MEK treatment for 48–72 h; in case of a suspected COVID-19 infection, a molecular test for virus detection was performed.

In conclusion, our experience supports the continuation or beginning of TT in metastatic melanoma patients. Both careful patient management at the hospital and the strict monitoring of symptoms in case of suspected COVID-19 infection are warranted.
